# Small signal modulation of photonic crystal surface emitting lasers

**DOI:** 10.1038/s41598-023-45414-7

**Published:** 2023-11-03

**Authors:** Jonathan R. Orchard, Pavlo Ivanov, Adam F. McKenzie, Calum H. Hill, Ibrahim Javed, Connor W. Munro, Jeff Kettle, Richard A. Hogg, David T. D. Childs, Richard J. E. Taylor

**Affiliations:** 1 Vector Photonics Ltd, Block 4.05, West of Scotland Science Park, Glasgow, G20 0SP UK; 2 James Watt School of Engineering, University of Glasgow, Glasgow, G12 8QQ UK

**Keywords:** Lasers, LEDs and light sources, Semiconductor lasers

## Abstract

We report the small-signal characterization of a PCSEL device, extracting damping factors and modulation efficiencies, and demonstrating -3 dB modulation bandwidths of up to 4.26 GHz. Based on modelling we show that, by reducing the device width and improving the active region design for high-speed modulation, direct modulation frequencies in excess of 50 GHz are achievable.

## Introduction

The demand for higher data transmission speed has stimulated research and over the past few years, bit-rates exceeding 40 Gbps have been demonstrated for GaAs based VCSELs emitting at 850, 980, and 1060 nm^1–3^. However, there is an issue in achieving long wavelength devices due to the poor index contrast materials systems other than GaAs/AlGAs. The need for long transmission distance along with low fibre dispersion, point to 1310 nm as the optimal laser emission wavelength. This necessitates an alternative laser technology in order to achieve the desired data rates.

Edge emitting lasers have historically been advanced through a shortening of the laser cavity to operate at speeds of 2.5 Gbps, 10 Gbps, 25 Gbps and beyond. The necessity to make the cavity short to increase the relaxation oscillation (RO) frequency has led to a problem in cleaving and handling the laser bars during production. Simultaneously achieving high speed and high yield in production volumes with such small bar lengths has been difficult and few companies worldwide have qualified products, and prices have not scaled down as with commodity 10 Gbps components. A solution to this has been to include a passive waveguide as padding so that the bars are cleaved to a standard length, and the active laser portion can be scaled down. The output power is reduced with chip length and the padding waveguide reduces the achievable device density on the wafer, so increasing chip cost. For conventional laser didoes, if the photon round trip times are designed appropriately, an additional pole in the frequency response can partly mitigate the roll-off of the frequency response in order to exceed modulation rates in excess of 50 Gbps. These photon-photon resonance (PPR) devices^4^ are being developed on native InP^5^, or Silicon carbide^6^ substrates. Additional complexity in design and manufacture mean that they have not yet reached market and will come with a high cost overhead.

An alternate approach is the photonic crystal surface emitting laser (PCSEL). This device incorporates a grating layer patterned to give a two-dimensional variation in refractive index across the device and by utilizing 2nd order Bragg diffraction laser emission can be coupled out of the surface of the device. The surface emitting nature of the PCSEL allows for on wafer testing, along with simple VCSEL-like fabrication and no requirement for cleaving and coating of devices can result in very low-cost devices. Historically PCSELs have been formed by wafer fusion, but these devices suffered from low output powers due to defect states in the PC region as a result of regions of discontinuous crystallinity formed during the wafer fusion process^7–9^. More recently our group^10–12^ along with several others have demonstrated PCSELs formed by epitaxial regrowth^13–17^. The move to epitaxial regrowth by metalorganic vapour phase epitaxy (MOVPE) has demonstrated a pathway to high volume production of highly reliable devices leveraging all the knowledge gained over many years of InP DFB development. InP devices emitting at 1310 nm^18,19^ and 1550 nm^20^ have already been demonstrated using this approach. To date, PCSELs have demonstrated single mode operation^7^, low beam divergence^8^, polarization and beam shape control^9,21–24^, making these devices ideal candidates for coupling efficiently to single mode fibre without the need for costly beam shaping optics. Additionally, high power and brightness devices^13^ as well as coherently coupled arrays of devices^25^ have been demonstrated. Recently, an intrinsic modulation bandwidth up to 1.68 GHz has been reported for PCSELs^26^, and modulation rates up to 2 GHz have been confirmed for a PCSEL used in a 64 QAM modulation scheme^27^.

In this letter, we report the analysis of small signal modulation measurements for a PCSEL device operated at a range of currents above threshold. Modulation bandwidths up to 4.26 GHz are demonstrated, and a damping factor (K factor) of 0.35 ns and modulation efficiency (D factor) of 4.45 GHz/mA^1/2^ are obtained. Based on modelling, we show that by reducing the device width and improving the active region design for high-speed modulation, direct modulation frequencies in excess of 50 GHz may be achievable.

## Small signal analysis

**Figure 1:**
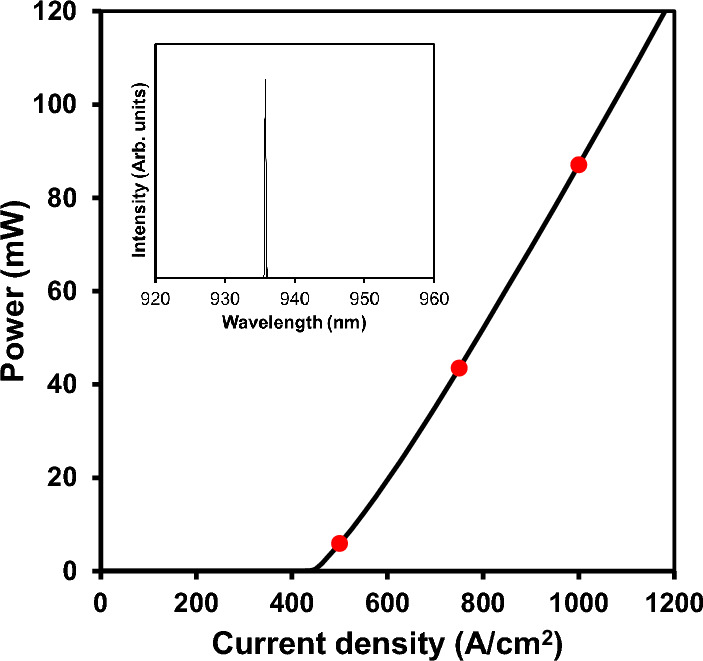
CW device output power for increasing current density. Solid points (red) on the LI curve show the bias currents at which the measurements in Fig. 2. were taken. Inset plots normalized laser intensity against wavelength at 2.2Ith.

**Figure 2:**
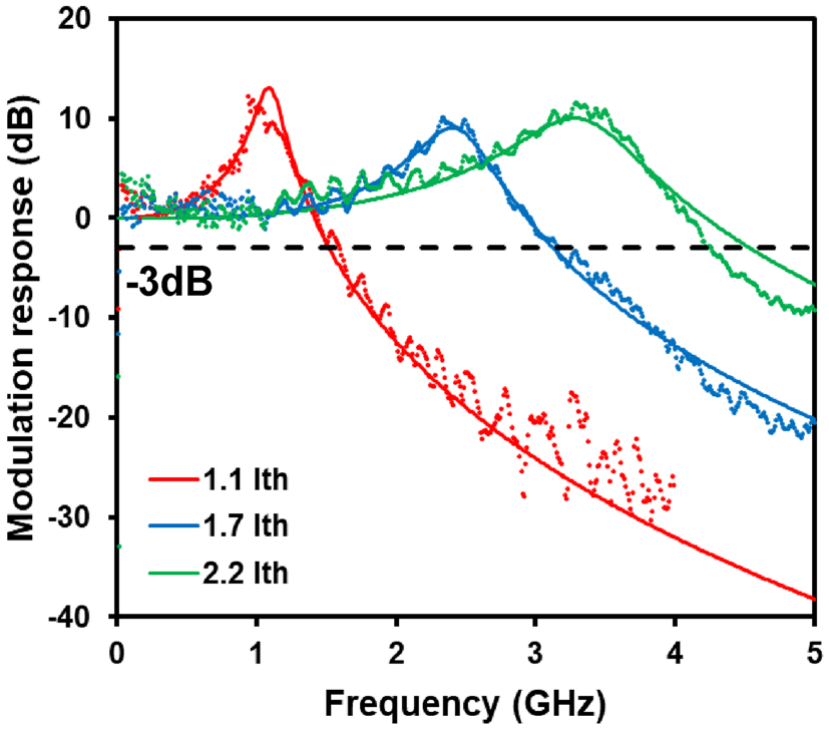
Small signal modulation response for the PCSEL at room temperature at three drive conditions of 500 A/cm^2^ (red), 750 A/cm^2^ (blue) and 1000 A/cm^2^ (green), corresponding to 1.1, 1.7, and 2.2 × threshold current, respectively. At 1000 A/cm^2^, a bandwidth of 4.2 GHz is achieved. The points represent the measured data with the solid lines showing the calculated fit to the data.

The room temperature, continuous wave output power as a function of current for the PCSEL device is plotted in Fig. 1. The GaAs-based device has a 200 × 200 μm square geometry, emitting at approximately 935 nm (as shown in the normalized emission spectrum, inset). The threshold current density (J_th_) of the device was 445 A/cm^2^ and the maximum measured output power was greater than 120 mW, corresponding to a wall-plug efficiency of 13.9%. The solid points (red) plotted on the LI curve in Fig. 1 correspond to the current densities, 500 A/cm^2^, 750 A/cm^2^, 1000 A/cm^2^ used for the measurements in Fig. 2. For spectral measurements, the device was driven at bias current of 2.2 × threshold and the light output was collected via a multimode optical fibre connected to an optical spectrum analyser.

Figure 2 shows the small signal modulation response for the PCSEL operating above threshold at room temperature, at the three drive conditions described above – these correspond to 1.1, 1.7, and 2.2 × threshold, respectively. Measurements were performed with R&S SMR20 signal generator at a power level of -10 dBm, Mini Circuits ZX85-12G-S + bias T to drive the laser, a focusing lens to match the detector area of a Newport Corporation 1437 M detector and amplitude measured by an Agilent E4440A ESA. Despite this being a large area device with contact area of 200 × 200 μm, optimised for high output power, a high -3 dB bandwidth of 4.2 GHz was achieved at room temperature for a bias of 2.2 × threshold. It should be noted that 100 mW class DFB lasers have similar modulation bandwidths^28^. Modulation bandwidths of 1.55 GHz and 3.13 GHz were obtained at 1.1 × and 1.7 × threshold, respectively.

**Figure 3:**
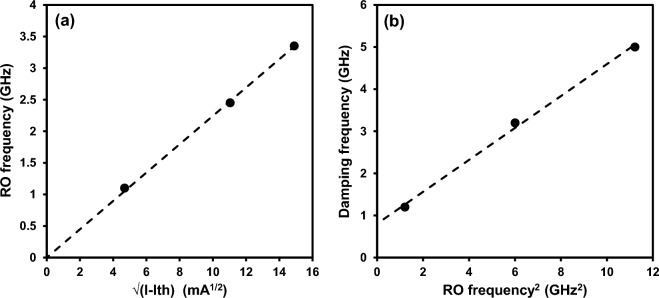
(**a**) RO frequency vs. square root of bias current density above threshold and (**b**) damping frequency (γ) as a function of RO frequency squared. Fitting to the data produces a modulation efficiency of 4.45 GHz/mA, and K factor of 0.35 ns.

From the data shown in Fig. 2, the relaxation oscillation (RO) frequency and damping frequency for the different bias currents can be extracted by fitting with the well-known equation, which is derived from a rate equation analysis of the electron and photon rate equations for a semiconductor laser^29^. Figure 3a plots the RO frequency (*f*_*R*_) as a function of square root of bias current above threshold (*I*_*th*_) for the PCSEL. We can clearly see a linear relation as expected from the increase in photon density in the cavity. The RO peak at 2.2 × threshold is 3.3 GHz, which corresponds to a power level of 87 mW, as shown in Fig. 1.

1 $$f_{R} = \frac{1}{{2\pi }}\sqrt {\frac{{{\delta g/ dn}\, \nu _{g} }}{{qV_{p} }}n_{i} \left( {I - I_{{th}} } \right)}$$

2 $$\gamma =K{f}_{R}^{2}+{\gamma }_{0}$$

Equations (1) and (2) taken from^29^ can be used to fit the data in Fig. 3(a) and 3(b) respectively, where *f*_*R*_ is the RO frequency, *δg/δn* is the differential gain, *ν*_*g*_ is the mode velocity, *η*_*i*_ is the internal quantum efficiency, *q* is the electron charge, *V*_*p*_ is mode volume, *I* is the bias current and *I*_*th*_ is the laser threshold current. For Eq. (2) *γ* is the damping factor, *K* is the ‘K factor’ and *γ*_*0*_ is the damping factor offset. The gradient of the line fit to the data in Fig. 3a using Eq. (1) is equal to the modulation efficiency or ‘D factor’, which gives a value of 4.45 GHz/mA^1/2^. Figure 3b plots the extracted damping rate (*γ*) versus RO frequency (*f*_*R*_) squared at room temperature. Again, a good approximation to a linear fit is observed, where the gradient is referred to as the ‘K factor’. For this PCSEL, the K factor is 0.35 ns. Using this value, the max 3 dB operation of the device can be calculated using f_3dBmax_ = √2 × 2π/K, this yields a damping limit to device operation of approximately 25 GHz. In practice the large metallization pad on these test devices limits the modulation frequency response due to RC parasitic effects to < 10 GHz. With small cavity edge emitting lasers, the K factor limit is rarely achievable since a significant junction temperature rise occurs when operated well above 10 kA/cm^2^. For the 100 mW PCSEL reported here, the 3 dB bandwidth thermal limit is expected to be < 20 GHz.

## Device modelling

**Figure 4:**
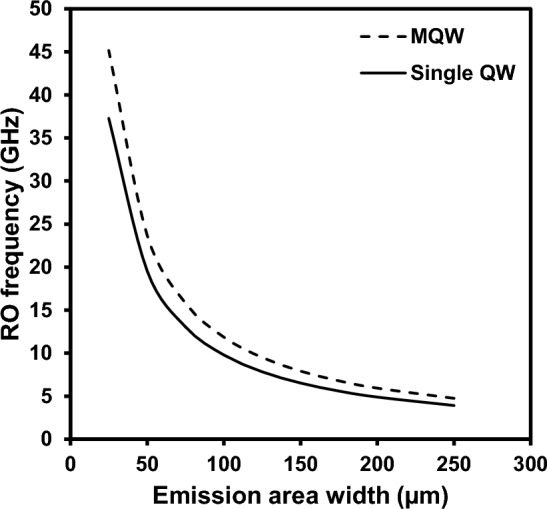
Calculated RO frequency at different device sizes for a single QW (solid line) and a MQW active element (dotted line). For a PCSEL with diameter of 25 µm, the maximum RO frequencies achieved are 37 GHz and 45 GHz, respectively.

As shown in Eq. 1, as the photon density in the cavity rises above threshold, the RO frequency increases, due to the exchange of energy between the electron and photon populations. The rate of increase is related to the design of the QW stack through the differential gain, and to the geometry of the laser through the optical mode volume. As such the RO frequency can be increased by decreasing the mode volume and increasing the differential gain. PCSELs are unique among semiconductor lasers in that the gain region can be designed in the same way as an edge emitting laser and hence are able to support an active region with a large number of QWs, resulting in a high differential gain. Whilst simultaneously being able to shrink to a small mode volume due to the vertical emission, allowing for two dimensions of optimisation to yield extremely high-speed direct modulation operation. Using the method laid out in^29^ we have calculated the RO frequency for a number of different device sizes using two distinct values for differential gain, this is plotted in Fig. 4. These RO frequencies were calculated using a constant value for in-plane loss, and constant J of 1 kA/cm^2^. A *δg/δn* of 1.5 × 10^–15^, is taken as the value given by a ‘single QW’ design^29^, and the ‘MQW’ value of 2.2 × 10^–15^, is that given by a state-of-the-art high speed edge emitting laser active region design with a large number of strained QWs^30^. As can be seen for both values of gain used the RO frequency increases dramatically as the width of the device becomes small, with a 25 µm diameter device giving a RO frequency of 37 GHz and 45 GHz for the current and improved differential gains, respectively.

It is noted at this point that, for conventional PCSEL designs, in-plane losses are expected to increase as PC width is reduced and may become prohibitively high, preventing lasing being achieved. However, a number of approaches exist for reducing these losses, such as the inclusion of a perimeter mirror^31^ or photonic crystal heterostructure^32^, reflecting light back into the cavity. It is expected that such approaches would allow for a significant reduction in active device size and the fabrication of devices tens of microns in diameter with reasonable threshold gains. However, these theoretical reports have yet to be realised practically, providing a new research challenge for PCSEL design and manufacture. The initial and optimised values for the modulation response of PCSELs determined in this work compared with the corresponding values for contemporary classes of laser are shown in Table 1, highlighting the potential for achieving high-modulation rate devices > 40 GHz^28,33–36^. To date, reports on the operating parameters of InP based PCSEL are comparatively scarce as compared to GaAs, having been initiated much more recently^18–20^. However, essentially identical approaches in terms of fabrication and design have been observed, suggesting that the results presented here indicate prospects for high-modulation rate surface emitting lasers at fibre-optic communications wavelengths.

**Table 1:** Comparison of initially reported and optimised (i.e. state-of-the-art) values of modulation response for DFBs^28, 33, 34^, VCSELs^35, 36^, and PCSELs (as determined in this work).

Laser class	Modulation response (GHz)			
	Initial reports	References	Optimised reports	References
DFB	2.5–4.5	^33^	15–25	^28, 34^
VCSEL	3.0–8.0	^35^	20–35	^36^
PCSEL	1.55–4.26	Measured	> 40	Calculated

## Conclusion

We have reported the analysis of small-signal modulation measurement for a PCSEL device. The large area device, not optimized for high-speed operation, exhibits a -3 dB bandwidth in excess of 4.2 GHz at 90 mW of output power. Based on the extracted K-factor, the device has a theoretical maximum -3 dB operation frequency of 25 GHz, excluding parasitic and thermal effects. Modelling of the PCSEL device has shown that by reducing the device width and optimising the active region design for high-speed operation a RO frequency of 45 GHz can be achieved, resulting in maximum -3 dB direct modulation frequencies in excess of 50 GHz.

## Data Availability

The datasets generated and/or analyzed during the current study are available from the corresponding author on reasonable request.
